# Extracts and Gleanings

**Published:** 1873-07

**Authors:** 


### PART II.

### EXTRACTS AND GLEANINGS FROM OUR EXCHANGES.

### *MULTUM IN PARVO.*

Ferric Alum in Purpura Hemorrhagica.—Ellen W., aged five years, was brought to my office on the 15th of April last, presenting all the symptoms of purpura hemorrhagica, viz.: purple or bruised-looking spots all over the face, neck, breast, arms, body and inferior extremities, with some abrasions upon the latter; hemorrhage from the ears, nose and gums. The whole skin, except the parts presenting the purple patches, was almost of an orange hue. These symptoms were said to be of only a few days' standing, up to which time her health was supposed to be good. The family had recently removed to this vicinity, and are unknown to me, but I am satisfied that there is scrofulous and syphilitic taint in it.

Powdered ferric alum was ordered, in doses of five grains, in simple sirup, three times daily. The hemorrhage ceased immediately. The case was presented on yesterday, all trace of the affection having passed away—the skin clear, ruddy and healthy to all appearance. No other remedy was used in the treatment of the case. I will not crowd your pages by offering any theory or reason for using the remedy. A. G. Smythe, M.D.

Calabar Bean.—Within the last six months several cases of tetanus, traumatic and idiopathic, have been treated with the calabar bean, in this city, with satisfaction. The saturated tincture was used, by mouth and hypodermically. When used in the former way, five drops in water were given every three hours, and increased when necessary. When in the latter, one drop in solution every two hours, and increased to meet the requirements. Although two cases died, it exerted a marked influence upon the spasms; and it is believed that, had the circumstances been favorable to the regular and skilled use of the drug, every case would have recovered. The physicians who have used the calabar bean feel that it is the Hercules of the Materia Medica in trismus.

E. T. Henry, M.D.

Propylamin Revived as a Cure for Rheumatism.—Twenty years ago propylamin was introduced as a remedy for acute rheumatism, and as such enjoyed some popularity. But its career was brief, and the remedy was soon forgotten. At that time the present writer gave it a trial, and found nothing to commend it but its unsufferable nastiness, which was so great that some patients preferred rheumatism to propylamin. Of late, it has been revived as almost a specific. At the sessions of the "Medical Society of the Hospitals," in Paris, (vide *Gaz. Hebdomadaire*, January 17, 24, 31), a number of practitioners asserted its efficacy, and reported numerous cures of acute rheumatism in a few days. Others, however, made opposite reports, based on their experience. M. Champouillon doubted whether acute articular rheumatism could ever be cured in ten days. The question was left for further experimentation. In regard to the preparation of the agent, M. Dessaigne had made from it a fixed, crystallized salt, of invariable composition, the chlor-hydrate of trimethylamin, the therapeutic use of which would be more commodious if its action should prove identical. Three specimens of this salt were exhibited, one made from herring pickle, one from Chenopodium vulgare (wormseed), and the third from human urine. The best mode of administering propylamin is in capsules, or dragees, each containing about a grain. *Pacific Medical and Surgical Journal.*

Chloral Hydrate in Puerperal Convulsions.—Edward Montgomery, M.D., of St. Louis, Missouri, (*St. Louis Medical and Surgical Journal*), after employing venesection and administering brisk purgatives, in cases of epileptiform or apoplectic puerperal convulsions, then greatly prefers the chloral hydrate to the chloroform, or the ether and chloroform treatment. He thinks that the chloral is much safer, administered in fifteen or twenty-grain doses, and it can be given safely and efficiently by any sensible and experienced nurse. In administering this remedy, he gives it in a dilute solution, and never repeats the doses at too short intervals. He generally orders, every two or three hours, two drachms in four ounces of spearmint or cinnamon water; a tablespoonful of this solution in a tablespoonful of sirup for a dose.—*New York Medical Record.*

Formula for Podophyllin.—A writer in an exchange says: "The griping effects of podophyllin resin may be readily obviated by combining it with small doses of extract of hyoscyamus. You will find the following a good formula for podophyllin pills, sometimes sold under the name of "Aperitive Seeds," or "Castor-oil Pills:" ℞. Res. podophyll., ext. hyoscyam., aa. grs. iij.; sapon. dur., grs. ivss.; sirupi, gtts. vj. M. ft. pil. xii. in arg. fol."

Acne Rosacea.—Dr. F. D. Weisse thinks that this disease is frequently dependent upon sexual difficulties, and is often cured by marriage. He agrees that digestive disturbances are also a potent cause. Young ladies thus affected, he has observed, as a rule, avoid fat and eat but little meat. The use of these should be encouraged, and fatty matter may be added to the diet or given as medicine, as half a pint of cream daily, glycerin, cod-liver oil, or even olive oil. As local applications, Dr. W. advises any greasy application, even simple suet, scented, to be applied at night after the hot douche, followed by squeezing the masses from the follicles. In the morning the fatty matter is still left on, the face not washed, but a powder of equal parts of subnitrate of bismuth and prepared chalk is dusted on, and removed by lightly brushing the face. Hebra employs a very weak ointment of the bichloride of mercury. The speaker had also used an ointment as follows: ℞. Pulv. camph., gr. v.; sulph. flor., ʒ ss–i.; ung. simpl., ℥ i. M. He considers acne rosacea thoroughly amenable to treatment. He had pressed arsenic to a toxic effect, and kept it up for six weeks, without any benefit in this disease. He had seen, however, good effects from cod-liver oil.

Vehicle for the Internal Administration of Chloroform (*Journal de Pharm. et de Chimie*).—Dr. G. W. Murdonck, having tried various formulæ proposed to facilitate the ingestion of chloroform, has found that some were difficult of execution, others contained sulphuric ether, and others again contained but little chloroform. He considers the best proceeding to consist in dissolving the chloroform in glycerin (1 : 3), which is effected with tolerable facility, and gives a very clear solution, pleasant to the taste, and with a strong odor of chloroform. This solution can be mixed in all proportions with water, without the occurrence of any precipitation, though the odor is distinctly perceptible. In forming the mixture, it is well to add the chloroform slowly, and to mingle the two thoroughly. It should be left at rest for twenty-four hours; at the expiration of this period, a portion of the chloroform will be found to have been collected at the bottom of the vase; this should be separated and mixed with an additional part of glycerin, when no further separation will occur. This mixture may be kept for some time without any loss of chloroform by evaporation.—*Medical Times.*

Dr. Hanot recently reported to the *Société de Biologie* in Paris a case in which uræmia, supervening on general paralysis, was diagnosed by the symptom pointed out by Bourneville—a rapid and general decline of temperature.

On the Use of Phosphorus in Certain Diseases of the Skin (*Dublin Journal of Medical Science*).—It appears that Dr. Burgess was the first physician who recommended the use of phosphorus in certain skin diseases, but Dr. Broadbent has recently given the same metalloid with good effect in some cases, and Dr. Tilbury Fox has also recommended its use; but Dr. Eames considers that phosphorus is much more than a substitute for arsenic, as some previous writers have regarded it, and he alleges that it has been used with marked success in certain cases in which arsenic has failed. The mode of administration adopted by him was a solution of the phosphorus in oil, and the dose of the solution was from five to ten minims three times a day after meals. Dr. Eames relates the particulars of several cases which were thus treated, and in all the results were successful. One was an instance of acne indurata of a most severe character, which had resisted all other local and general treatment; three of the cases were instances of lupus; and two of scrofula-derma. In psoriasis Dr. Eames found phosphorus very efficacious, even when arsenic had proved unserviceable, and he gives three cases in proof of this statement. A case of pemphigus was also cured by the use of phosphorus. Dr. Eames found that the drug produced a coated state of the tongue, and sometimes symptoms of dyspepsia, loss of appetite, mental depression, and bodily weakness; but, when these symptoms appeared, the phosphorus was discontinued for a time, and mineral acids substituted. Most of the cases recorded by Dr. Eames had been treated by arsenic and other drugs before they came under his care, and he regards phosphorus as far superior in efficacy to that and other vaunted specifics in skin diseases.—*Half-Yearly Abstract.*

How to Deprive Iodine of its Stain.—Add a few drops of phenic acid to the tincture, and it will not stain; moreover, the tincture is more efficacious, and its action more certain. M. Bogs recommends the following formula for use in injections: Alcoholic tincture of iodine,, three grm.; phenic acid, six drops; glycerin, thirty grm.; distilled water, one hundred and fifty grm. This preparation is superior to all others in the treatment of blenorrhagia and leucorrhœa.—*France Medicale.*

New Anodyne Colloid.—The *London Practitioner* gives the following formula as likely to prove a useful application in neuralgic and other localized pains: ℞. Amyl. hydrid., ℥j.; collodion (B. P.), ℥j.; aconitiæ, gr. i.; veratriæ, gr. vj. This forms a colloid which should be brushed over the painful part five or six times, forming successive films. If the pain be not at once relieved, the absorption of the alkaloids may be favored by covering the colloid film with a layer of spongio-piline.

TOOTHACHE.—In nervous irritability dependent on continuous toothache, what is the best treatment? Few remedies act better on the languid, nervous individual than hydrate of chloral; to prescribe it, the following makes a satisfactory formula: ℞. Chloral hydratis, ℥ss.; sirupi tolutanis, aqua, aa. ℥iij. M. Dose from two teaspoonsful to a tablespoonful. If the pulse be flagging, it is well, however, to precede the exhibition with a glass of wine or other stimulant.

Another and a very admirable plan of treating such irritability is found in the use of the electro-galvanic current. The feet and hands of the patient are to be put in basins of milk-warm water; in the basin holding the feet, place the positive pole; in that containing the hands, the negative.

When odontalgia is associated with general vascular disturbance, few remedies act to more advantage than full doses of the bromide of potassium conjoined with veratrum viride, from twenty to sixty grains of the first, dissolved in half a goblet of water, and from three to eight drops of the tincture of the latter. A hot pediluvium, where there is special symptom of head congestion, proves a valuable adjunct.—S. P. G., *Dental Cosmos.*

STYPTIC COLLODION.—℞. Tannin, ℥ij.; stronger alcohol, f℥iv.; stronger ether, f℥xij.; soluble cotton, ʒj., ℈ij.; Canada balsam, ʒj. Introduce the cotton into a suitable bottle, pour on it two fluid ounces of alcohol, shake well; then add ten fluid ounces of the ether, agitate frequently until dissolved. Dissolve the tannic acid in a mixture of the remainder of the alcohol and ether, mix with the first liquid, add the balsam, allow to stand until clear; then pour off.

*Collodion with Sesquichloride of Iron.*—℞. Sesquichloride of iron, ʒj., grs. x.; stronger alcohol, f℥x.; stronger ether, f℥xij.; soluble cotton, ʒj., grs. x. Into a soluble bottle introduce the cotton, pour on two fluid ounces of the alcohol, and shake well; then add the ether, and agitate frequently until dissolved. Dissolve the sesquichloride of iron in the balance of the alcohol, mix with the prepared collodion.—*American Journal of Pharmacy.*

CASE OF BERIBERI.—An interesting case of beriberi, the symptoms of which at one time strongly resembled those of aortic aneurism, is detailed in the *Madras Monthly Journal of Medical Science*, by G. Williamson, M.D., Assistant-Surgeon of the 19th M. N. I. At last advices, the patient, private, aged 33 years, was on sick leave to his native village, and doing well. The formula most beneficial to him was the following: ℞. Potas. acet., grs. x.; tinct. scillæ, gtts. x.; spir. nitric ether, gtts. xx.; ammon. acet., ℥ij.; camp. mixt., ℥i. To be taken every three hours.

Gelseminum in Gynæcology.—Dr. H. V. Ferrell, Carbondale, Illinois, writes to the *Clinic*, that two doses of gelseminum, of ten drops each, entirely relieved the excessive irritability of the uterus in a case of endometritis, and the os, which was before the administration of gelseminum rigid and contracted, dilated. Another case—suppression of lochia, and retention of part of the placenta—the result of the stupidity of a midwife. Present condition of patient alarming; in which, on examination, the os was found firmly contracted, and hardly admitted the end of the index finger. The patient was treated with gelseminum and belladonna, and in twelve hours the os dilated so as to admit two fingers, and permit the extraction of the retained placenta, etc., when the patient recovered without further trouble.

Gelseminum (in five drops every hour, in water, till relief is obtained, and then at longer intervals,) has relieved after-pains in very severe cases. Dr. Shaw, of Guthrie, Iowa, claims to have had the best results from gelseminum in the treatment of uterine leucorrhœa, and Dr. Mobsworth, of Des Moines, places more reliance in it for the successful treatment of chronic dysmenorrhœa than in any other remedy. Both these gentlemen say its good effects in these affections are frequently as prompt and decided as is quinine in intermittents.—*Medical Archives.*

Chronic Conjunctivitis in Children.—Tinct. of aconite root, ℥ss.; water, ℥iv. Three or four drops are instilled into the eye occasionally during the day.—*Eclectic Medical Journal.*

Atropia, gr. j.; tinct. verat. vir., ʒss.; water, ℥j.; in severe cases where the light causes pain, and the cornea injected. Use as above. Old and obstinate cases will be highly benefited by the acetates of potash and ammonia, in conjunction with fluid extract of cinchona.

℞. Potass. acet., ℥ij.; water, ℥j.; glycerin, ℥j.; fld. ext. cinchona, ℥ss.; teaspoonful every four hours is a good formula.—*Medical Archives.*

Treatment of Hooping-Cough.—Dr. Charles Kelly, in the *Practitioner* for March, 1873, reports some experiments with belladonna in this disease. It was given in full doses, gtts. x. to ʒss. of the tincture every two, three or four hours for four or five days at a time; and the effect seemed to be to lessen the severity and duration of the coughing spells.

Dr. Berry, of Lancaster, is quoted in the same journal as advocating the use of dilute nitric acid, gtts. v. to xv., given in simple sirup every three or four hours. He thinks it not only allayed the cough and spasm, but actually cut short the disease.

Oulmont on Treatment of Mercural Tremors and Paralysis Agitans by Hyoscyamine.—Dr. Oulmont (*Gazette des Hôpitaux*, January 11, 1873), considers that, although the results obtained by the treatment of neuralgia by hyoscyamine are satisfactory, they are neither more marked nor more rapid than those obtained by the use of opium or belladonna. In nervous diseases characterized by trembling, such as mercurial tremors and paralysis agitans, marked benefit attends its employment, relief or cure being effected by it after all the other usual remedies, such as iodide of potassium, tonics, electricity, sulphurous baths, bromide potassium, opium, etc., have failed. Four cases of tremors, treated with hyoscyamine, were cured, and two relieved; and two cases of paralysis agitans were relieved. In mercurial tremors he begins with a dose of two or three milligrammes of hyoscyamine daily, and gradually raises it, notwithstanding dryness of the mouth, etc., to twelve, or even seventeen, milligrammes daily. As the trembling ceases, the dose is gradually diminished. In paralysis agitans he gives six milligrammes daily, at first, and gradually raises the dose to ten.

Pulv. Rhei Co. Granulata.—The London *Chemist and Druggist* highly recommends a preparation under the above title, intended to render tasteless "that valuable but nauseous compound, so long celebrated as Dr. Gregory's Stomachic Powder, officinal in the British Pharmacopœia as Pulv. Rhei Co." The mode of preparing it is as follows: "Take any quantity of pulv. rhei co.; mix in a mortar with sufficient sirup to form a moderately tough mass (the proper consistence will be easily determined after one or two experiments), pass through a brass sieve, and allow it to remain ten or twelve hours to dry; then coat with tolu dissolved in chloroform. I find the easiest method of coating is to nearly fill a two-ounce chip-box with the dried granules; pour in about ʒss. of tolu solution; agitate briskly, and pour out the contents of the box upon a sheet of paper. Expose them to the air for an hour or two, and they will be fit for use."—*Boston Journal of Chemistry.*

Corneal Affections—Dr. Williams's Prescription.—Atropine is well known as a good remedy in all kinds of corneal affections. It is, therefore, to be used in small-pox keratitis. Carbolic acid should be combined with it. In this combination, in my judgment and experience, we shall have the best known remedy for small-pox keratitis. The following is the form of prescription I always use: ℞. Atropine sulph., gr. iv.; acid carbolic, gr. v.; aqua distillat., ℥j. Mix. To be dropped into the eyes three to five times a day, according to severity of the keratitis; two or three times at night, if there is much pain.—*Medical Archives.*

Little on the Use of Digitalis in the Failing Heart and Delirium of Acute Diseases.—Dr. Little (*Irish Hospital Gazette*, January 15, 1873), recommends the use of digitalis in febrile cases in which stimulants are either not well borne or are contra-indicated, as, for example, in most cases where renal affection is present. He has given it in more than twenty cases, including six of typhus and one of rheumatic fever, the remainder being cases of enteric fever. He usually gives half drachm doses of the tincture every three or four hours, rarely every hour, and discontinues the medicine when the pulse falls to eighty. Along with the digitalis he gives wine and brandy. In one case of rheumatic fever, where stimulants could not be borne, he used tincture of digitalis alone. After several doses nausea occurred. The tincture was, therefore, discontinued, and a hypodermic injection, composed of one-eightieth of a grain of atropia, one-fortieth of a grain of digitalin, and one-fourth of a grain of morphia, was substituted The patient eventually recovered.

Local Employment of Chlorate of Potash in Cancerous Sores (*Lancet*, April 12).—In the *Berl. Klin. Wochenschrift*, No. 6, 1873, Dr. Burow, of Königsberg, advocates the local employment of chlorate of potash in the treatment of cancerous sores. His proceeding consists in sprinkling the sore with chlorate of potash in powder or crystals, and covering the whole with a wet compress. As the crystals of chlorate of potash exert a more powerful action than the powder, and excite greater pain, Dr. Burow uses the powder first, and replaces it by the crystals when sensibility has been abated. One of the cases was a cancerous sore of the left arm, which healed completely after eight weeks' treatment. Three other cases were cancerous sores of the breast: one was lost sight of, the other two are under treatment and healing well. The fifth case recorded was connected with a cancer which originated in the periosteum of the upper jaw and cheek-bone, and then became ulcerated; in this case healing was complete in three months.

Boils, their Cause and Cure.—Dr. J. D. McGaughey, in *Medical Times* (March 8), gives some good reasons for thinking that the probable cause of boils is a temporary increase of the white blood corpuscles. For their cure he recommends the use of quinine in doses sufficient to affect the head, and continued till recovery. Arsenic he thinks the next best remedy. This view is further supported by the observations of Binz. He found that quinine kills the white blood corpuscles, and when given by the mouth the quantity of white blood corpuscles in the blood was diminished.

TREATMENT OF WARTS.—In a lecture in the *Medical Times and Gazette*, London, Mr. J. W. Hulke draws the following conclusions from the cases that have fallen under his notice: 1. Not to think too lightly of small, hard pimples and warts in the face, in persons advanced in life, but promptly and thoroughly to excise them, especially if they show a tendency to grow, ooze and scab. Their complete excision, together with a broad fringe of sound tissue, will probably secure a future immunity. It is advantageous to close the fresh wound, when large, with a flap of sound skin from an adjoining part. 2. Never to try to destroy them with nitrate of silver, as is too often done, because its action is too superficial, and its use seems often to hasten their progress. 3. When the surface of the ulcer is so irregular that complete excision is impracticable, it should be supplemented by freely burning with a hot iron those parts which could not be reached with the knife, and by the free application to them of the chloride of zinc paste. 4. Some apparently desperate cases at an advanced stage may thus be treated very successfully, if the patients possess confidence and docility. 5. Such composite operations are well borne by persons in advanced years, if care be taken to avoid much bleeding.—*Medical and Surgical Reporter.*

LIQUOR PICIS ALKALINUS.—This preparation was originally devised by the late Dr. H. D. Bulkley, of New York, to fill the place of a secret French preparation of tar. Dr. L. D. Bulkley, in the February number of *Archives of Scientific and Practical Medicine*, gives the formula thus: ℞. Picis liquidæ, ℥ ii.; potassæ causticæ, ℥ i.; aquæ, ℥ v. M. ft. sol. This mixes with water in all proportions, and discolors the skin to a very moderate extent. It dries rapidly, and leaves very little stickiness. He has used it in all degrees of strength, and regards it as one of the best methods of employing tar. The potash heightens the antipruritic effect of the tar. The solution he has employed with advantage in eczema, both in its chronic stage with thickenings, and in the more acute forms, where exudation has about or nearly ceased and the itching is intense. In chronic cases with infiltration, it may be used in full strength. Good success has followed its use in lupus erythematosus and psoriasis.—*Detroit Review of Medicine.*

CEREBRO-SPINAL MENINGITIS.—Professor Loomis, of New York, who has considerable experience in the treatment of this affection, gives the following: Sol. saturat-potass. bromide, forty minims every two or three hours; quinia sulph., three to five grains every three hours; ice to the head and spine; blisters to the nape of the neck; bleeding, when the constitution of the patient will admit of it, and tonics during convalescence.

New Operation for the Radical Cure of Hernia.—A Spanish Professor, Dr. Egea, of Madrid, struck by the idea that failure was due to incomplete invagination, has adopted a procedure by which he performs perfect invagination. For this purpose he makes use of an ordinary thimble, having a hole at the end and a lateral groove in which a transverse bit of steel may be moved. Hernia being reduced, invagination is effected with the left index; a long, lance-like needle, with a strong thread passing through the hole in the thimble, is introduced upon the index, and is thus carried from within outwards, through the fundus of the invaginated sac, the neck of the hernial sac, and the abdominal walls, as high in front and externally as possible, so as to avoid wounding the internal organs. The needle thus draws upward the thread, whose other extremity is attached to the bit of metal placed transversely in the thimble; this, dragged upwards, gets into the invagination, and takes the place of the index. The thimble is then secured in its position by means of a suture, whilst the thread is attached to an immovable bandage round the body.—*Medical and Surgical Reporter.*

Night Sweats of Phthisis.—For the relief of this exceedingly troublesome symptom in this disease, a comparatively new remedy is being used somewhat. It is the sulphate of atropia. Some of the patients are taking one-sixtieth of a grain in solution *ter in die;* some are taking one-hundredth of a grain at bed-time, and all are giving very good results. The success in this direction has already been sufficient to entitle it to a trial at least. A very excellent plan of managing these night sweats consists in sponging the patient in hot water, as hot as they can comfortably bear. If a patient is found sweating profusely in the night, take him out of bed, sponge him thoroughly with hot water, wipe him dry, replace his flannels, and put him back to bed. Sometimes a single sponging will arrest the sweating for two or three days.—*New York Medical Record.*

Eczema.—Dr. C. O. Wright reported a case of eczema which had for some time resisted the ordinary means of treatment. He accidentally discovered that the patient had been using Brown Windsor soap. Not being able to assign any reason for the long continuance of the disease, he concluded to try the effect of discontinuing the soap. This was followed by an immediate abatement of the disease. Not considering this as positive proof of the agency of the soap in causing the disease, he resumed the soap and the eczema reappeared. Discontinued it and continued his former medication, and the case was soon cured.—*Clinic.*

DETECTION OF INDICAN IN THE URINE.—Among the rarer abnormal constituents of the urine, indican deserves more attention than it has heretofore received, if, as Dr. Neftel asserts, its presence in large quantities is evidence of the extension of carcinoma to the internal organs. Indican in the urine is most readily detected by the method of Jaffe. The urine is mixed with an equal volume of hydrochloric acid, to which a few drops of concentrated solution of chloride of lime have been added. The liquid at once assumes a blue color, and forms, after awhile, a sediment of indigo blue. If the quantity of indican is small, the urine must first be concentrated by evaporation over the water bath. During the evaporation it should be kept alkaline, to prevent decomposition. When it is reduced to a sirupy consistency, it is to be treated with strong alcohol, filtered after twenty-four hours' standing, and finally freed from alcohol by distillation. The alcoholic extract is then dissolved in water, precipitated with chloride of iron, filtered, and the excess of iron precipitated by boiling ammonia and removed by filtration. This filtrate, evaporated to one-fourth of the original volume of the urine, will now give the reaction for indican as above described. *Detroit Review of Medicine.*

SIREDEY AND CORDIER ON THE TREATMENT OF ANASARCA, ASCITES, AND OBSTINATE PLEURITIC EFFUSIONS BY MILK.—MM. Sirédey and Cordier (*Journal de Medicine et de Chir. Prat.,*) have used this old and rather favorite remedy against dropsies in a way which they consider renders it less troublesome and repugnant than the "milk-cure" is otherwise apt to be. At the same time that Sirédey prescribes two pints of milk a day to his patients, he allows other kinds of food and drink. Cordia, in his thesis, cites cases of albuminuric dropsy in which the diuresis was rapid and considerable, and the dropsy disappeared under this treatment. The diet is otherwise moderate, including meat and a little water. He does not give more than two or two and a half pints of milk, at convenient intervals, in the day. He prefers asses' milk.—*Medical Times.*

NATURE AND TREATMENT OF CHOLERA.—As patients with cholera exhale an odor of pyroacetic ether or acetone, Sainmont believes that cholera is a sort of acetonemic poison, and the indication in treatment is to combat this toxicæmia by alkaline chlorides. The best method of practicing this treatment is by clysters of hot water containing a tablespoonful of common salt. This is to be repeated if not retained.

This treatment is not applicable to the choleraic form of diarrhœas.—*Lyon Medicale.*

THE EMPLOYMENT OF PESSARIES.—John Lambert, M.D., Salem, New York, (*Journal Gynæcological Society*), in a communication on "Uterine Displacements," says that the use of pessaries in these cases complicates treatment and hazards successful results. In a certain proportion of cases of uterine displacements, a comparatively small number of well-fitting pessaries, in the hands of intelligent and skillful gynæcologists, are essential to the cure of not only the mal-position, but also of the abnormal condition of the organ which accompanies it. A pessary of whatever kind, when employed for the mechanical support of the uterus, is a foreign body, liable to do serious and perhaps fatal mischief, and never should be placed *in situ* without great circumspection on the part of the physician. It has no miraculous power, and its potency for harm is very much underrated.

He is of the opinion that intra-uterine or stem pessaries should seldom be used. Of the various forms of intra-vaginal pessaries in use, he prefers and employs the closed lever made of hardened rubber, and the soft elastic ring made of delicate stips of fine whalebone, covered with pure rubber, made by Tieman & Co.—*New York Medical Record.*

INSARD ON THE TREATMENT OF CONSTIPATION BY ARSENIC. Dr. Insard (*Marseille Medical*, 1872) states that many other physicians besides himself have observed that arsenic excites the appetite and improves the digestion, and facilitates the action of the bowels, but he is not aware that it has been methodically applied for the relief of constipation. Besides (1) improving the appetite, it (2) excites the muscular action of the intestines, and (3) it augments the secretions of the alimentary mucous membrane. It is particularly useful in the constipation of debilitated and anæmic females, sedentary persons and old people. He administers arsenious acid in the dose of six to ten milligrammes twice or thrice a day, with food. Sometimes smaller doses suffice. It may be used steadily for some time.

TREATMENT OF SMALL POX BY USE OF TINCT. FERRI CHLORIDI.—In the *Medical Herald*, of March, we find an article from Dr. J. P. Rood, of Santiago, Chili, strongly recommending the use of tincture of iron in small pox. He gives it in all stages of the disease in doses of from five to fifty drops every four to six hours. Between the doses he gives from one-third to two teaspoonsful of prepared chalk. If warm, disinfectants are employed, and the ordinary hygienic measures. He believes that by the above plan he destroys the poison of the disease, and supports the patient. Thus the disease is cut short, and the patient cured in a week or less.—*Detroit Review of Medicine.*

Carbolic Acid in Skin Diseases.—A lady, aged about 35, who had undergone much anxiety and fatigue in nursing a sick child, was attacked for the first time with psoriasis. The patches, on an average about the size of florins, were of a dusky red tint, but not scaly. They were very numerous, especially upon the arms and legs. She felt weakly and was incapable of much exertion, and the feeling of fatigue and lassitude was very marked at times; otherwise her health was good. She was subjected to an arsenical course by her medical attendant, but without benefit. She then consulted Dr. Anderson, when, the arsenical course having failed, four grains of carbolic acid in solution were prescribed thrice daily. There was immediate improvement. The feeling of lassitude and exhaustion diminished, the eruption began to fade, and in a few weeks she was quite well.—*Dr. J. McCall Auderson, Half-Yearly Abstract.*

Neuralgia.—One of the best methods of treating neuralgia is that recommended by Frosseau [Trousseau ?] A tight top thimble is filled with cotton wool, and a few drops of strong aqua ammonia dropped on it. The open mouth of the thimble is then applied over the seat of pain for a minute or two until the skin is blistered. The skin is then rubbed off, and upon the denuded surface a small quantity of morphia (gr. ¼) is applied. This affords almost instant relief. A second application of the morphia, if required, is to be preceded by first rubbing off the new formation that has sprung up over the former blistered surface.—*Medical Archives.*

Treatment of Consumption at the Brompton Hospital. T. G. Roddick, M.D., House Surgeon to Montreal General Hospital, writing from London to the *Canada Medical and Surgical Journal,* says that two thousand gallons of cod-liver oil are consumed in that institution in a year. Lead, turpentine and gallic acid are the sheet-anchors there in hemoptysis. Although the physicians are beginning to use ergotine hypodermically with moderate success, they do not depend on the latter remedy in severe cases.—*Medical Record.*

Chronic Eczema of the Extremities.—Dr. MacCallum, of the Montreal General Hospital, (*Canada Medical and Surgical Journal*), successfully treated an obstinate case of this affection, the patient, male, being forty-two years old, as follows: A solution of potassa caustica, gr. v., to water, ℥ ij., was used outwardly, being brushed lightly over the eruption once daily. The following mixture was ordered internally: ℞. Hydrarg. bichlor., gr. iv.; liq. arsenic, gtts. xv.; acid hydrochlor., dil., ℥ iss.; aquæ ad., ℥ viij. M. ℥ ss *ter die.*—*Medical Record.*

Orchitis Treated with Nitrate of Silver.—Dr. Morse (San Francisco Medical Society—*Pacific Medical and Surgical Journal*) wished to mention a treatment of orchitis which had been attended with remarkable success. A year ago Dr. Prevost had a case of troublesome acute orchitis, for which he employed a new treatment, with results so wonderful as to induce its repetition in other cases. He, Dr. Morse, adopted the treatment in the case of a gentleman who was unable to subject himself to the ordinary treatment. The swelling was large and hard, yet it speedily receded and was gone, and the patient well in four days. Three other cases terminated as happily. The treatment consisted in rubbing the moist scrotum thoroughly with the stick of nitrate of silver—nothing more. It might be well to apply a simple poultice afterward, as he had directed in this case.

Rheumatism, Cause and Treatment.—In the Chicago *Medical Journal,* April, 1873, Dr. Whitmire published an elaborate article upon rheumatism. His especial object was to call the attention of the profession to the views of the late Professor Mitchell. These were as follows: "Rheumatism is a constitutional disease, the inflammation being brought about, and maintained, in consequence of functional derangement of the spinal cord, which derangement is produced by either active or passive congestion, or, perhaps, inanition of that important appendage to the great centre of the nervous system." In addition to the ordinary treatment of rheumatism, the Doctor recommends the frequent application of cups to the spine, or an occasional blister. This not only relieves the pains, but, the Doctor thinks, renders the disease less migratory, less liable to affect the pericardium, etc.

Digitalis an Anaphrodisiac.—M. Gourvat, in the course of a paper published in the *Gazette Medicale de Paris,* on the action of digitalis, says: "When digitalis or digitalin is administered for some time to a man in full possession of sexual powers, these become gradually weakened, the propensities disappear, formation of the liquor seminis diminishes, and may at last cease altogether. The anaphrodisiac properties of the drug are the secret of its good effect in spermatorrhœa."

Oxide of Zinc for Night Sweats.—The most ancient and venerable remedy for night sweats is aromatic sulphuric acid, in infusion of cinchona, serpentaria or sage. The best of all remedies, however, is this: ℞. Oxidi zinci, gr. xxx.; ext. hyoscyami, gr. xv. M. f. pil. x. Sig. Take one at bed-time.—*Pacific Medical and Surgical Journal.*

USE OF ACTEA IN LUMBAGO AND RHEUMATISM.—The *Practitioner* for March 13 contains a report by Dr. Bartlett upon the use of actea in lumbago and rheumatism. He could not discover any symptoms or chain of symptoms that specially indicated its use. Yet, in the above-mentioned diseases its beneficial effects were very marked. The average of the patients was thirty-nine years. Out of twenty-nine cases observed, fourteen were suffering from lumbago, of whom eleven were cured; fifteen were suffering from chronic and subacute rheumatism, of whom eleven were cured. In the chronic cases the actea gave immediate relief. He gave the actea in the form of tincture, in doses of half a drachm three times per day. Unpleasant symptoms caused by the drug were noticed in six cases. These were giddiness, headache, nausea, vomiting and irregular pulse. They ceased on discontinuing the drug.

ANODYNE COLLOID.—I beg to send herewith a bottle of a new colloid, which I have prescribed for some time as a topical anodyne application. It has given me very satisfactory results in neuralgia, sciatica, lumbago, all muscular pains, etc. It relieves local pain for the time, and procures a good night's rest, while, of course, the internal treatment appropriate to each case is carried on. I should wish to see its usefulness more extended. It contains—Hydride of amyl, ʒj.; aconitia, gr. j.; veratria, gr. vj.; and ethereal collodion to ʒij. The amyl, by its rapid volatilization, often produces, almost instantaneously, the desired result; but, should the pain continue, the alkaloids can be brought into activity by applying a piece of moist spongio-piline over the collodion film. The amyl hydride is the only new ingredient; but I think the colloid is a clean and elegant preparation.—*M. H. Lackersteen, British Medical Journal.*

POTASSIUM CHLORATE IN CATARRH.—Leonard W. Sedgwick, M.D., calls attention to the immense value of potassium chlorate in catarrh. If taken early and taken frequently, it will stop many a cold. The best form is the lozenge, eight or ten or more in the twenty-four hours. It should be sucked very slowly, for its action is chiefly, if not altogether, local. It always quickly relieves the stuffing of the nose, the rawness of the throat, the thickness of the voice, and, if begun soon enough, speedily cures the cold.—*British Medical Journal.*

HEMOPTYSIS, GALLIC ACID.—Dr. Holden, in the *Medical Record*, recommends a solution of gallic acid, used with the atomizer; no particular quantity of the acid is adhered to as a formula. He says it is prompt and effectual, soothing to the cough, as well as arresting the hemorrhage.

PHOSPHORUS MIXTURE.—A very pleasant preparation of phosphorus, in the fluid form, is: Dissolve a grain of phosphorus in six drachms of chloroform, and add two ounces of good glycerin, and shake well. The glycerin forms a permanent union with chloroform, which dilution with water does not separate. Dose, a teaspoonful three times a day.

Hypophosphorus acid has not received that attention its merits entitle it to. As a remedial agent of the phosphorous family, it is one of the most reliable. The hypophosphorus acid, in solution, enters the economy, in combination with some of the fatty acids and glycerin combined, and forms a true nerve food and restorative. For children it is of decided advantage. The usual dose is from ten drops to ʒ ss., with an equal quantity of glycerin in half to an ounce of some aromatic water.—*Medical Archives.*

TREATMENT OF CHRONIC DISEASE OF THE BLADDER BY THE INJECTION OF HEALTHY URINE.—Dr. Clemens, of Frankfort, uses healthy urine containing the normal amount of uric acid, as an injection into the bladder in cases of catarrh. The organ should be first cleaned from any fetid urine, and washed out by the injection of tepid water; a healthy, young subject is then got to pass urine into the syringe, which has been previously warmed, and then it is injected into the diseased bladder. The best results are obtained where the urine of healthy boys and adults is used on alternate occasions. In one instance, a single injection of healthy urine sufficed to allay spasm, and in others the injection, used twice or three times a day, produced a most beneficial result.—*The Clinic.*

SALICINE IN OBSTINATE DIARRHŒA.—Dr. J. B. Mattison, of Chester, New Jersey, in the *Medical and Surgical Reporter*, writes that salicine has proven in obstinate diarrhœa a valuable remedial agent in his hand. He administers it in either pill or powder, but prefers the latter for children, in the dose of half grain every four hours, when under two years old. For adults he advises the following formula: ℞. Salicine, ʒj.; make into twenty-four pills; two pills every four hours.

BELLADONNA IN INTESTINAL INVAGINATION AND HERNIA.—D. Gallicie, *La France Medicale* (*London Lancet*), in a paper on belladonna, says it is the special medicament for intestinal invagination, strangulated or not, as also for strangulated hernia. It acts on both the spasmodic and inflammatory elements. In both cases, however applied, its first effect is to alleviate the intensity of pain, and to diminish and arrest the vomiting.

Convulsions in a New-Born Child from Alcoholism of the Nurse.—Such facts are not very rare, but attention should be drawn to them more freely. Convulsions of infants are always grave, and when caused by alcoholism on the part of the nurse, it is always easy to check them. M. Vernay reports an interesting observation. It was the case of an infant in convulsions, for whom for five days calomel, bromide of potash, baths, musk, belladonna, etc., had been prescribed in vain. At last, M. Vernay ascertained that its nurse was in the habit of drinking six-eight glasses of wine every day, and indulged also in the night. On suppression of this habit the convulsions ceased.

Leroy, former professor of the faculty in *le Midi*, used to say that when a nurse wanted to put her baby to sleep easily, she drank freely of wine or brandy before giving the child the breast.—*Mouvement Medical.*

Ethiops Mineral in Cholera.—The *Medical and Surgical Reporter* says that Dr. Socrates Cadet, Professor in the University Royal, of Rome, has in a pamphlet, 1872, declared that ethiops mineral is a specific for Asiatic cholera, and produces numerous examples from the hospitals of Rome in support of this. As a preventive, he advises it in doses of four grains several times a week, and twenty to thirty grains several times repeated as a curative. "The extraordinarily favorable results he claims to have had with this drug certainly ought to secure for it a careful trial elsewhere." It has been discarded from the United States Pharmacopœia. So we go.

The Pulse in Childhood.—Mere irregularity of pulse is of absolutely no value as a means of diagnosis. In any febrile attack the pulse of a young child may become irregular; indeed, in many cases the mere quickening of the heart's action seems to lessen the regularity of its contractions. But while a *quick* irregular pulse is of little consequence, a *slow* irregular pulse carries a far different meaning, and, if suspicions of tubercular meningitis have been already excited, will go far to convert those suspicions into certainty.—*Smith, Medical Times and Gazette.*

Chloroform in Lead Colic.—Dr. Laramée reports a case of saturine colic, in which, after all other methods had failed, the application of a flannel over the abdomen, soaked in two ounces of chloroform, and over this a flannel wrung out of hot water, arrested the pains instantaneously and permanently. A light purgation finished the cure. He had a like result in a second case.—*L' Uuion Medicale.*

Mercury in the Treatment of Bronchitic Asthma.—Dr. Thorowgood, of the Hospital for Diseases of the Chest, observes that ordinary spasmodic asthma is a spasmodic neurosis of the lungs, and may, even in the most severe cases, be quite independent of any inflammatory or organic change in the pulmonary structures. Hence it is that we often get excellent cures by the employment of medicines of the nervo-tonic class, such as iron, quinine, arsenic, silver and zinc, with belladonna, stamonium, datura, tatula, etc.

In dealing with the complaint which he calls bronchitic asthma, a different plan of treatment is required, and he believes an important medicine in real bronchitic asthma is found in mercury. To illustrate the remedial action of this drug, he appends short notes of several cases in which mercurial treatment was employed with success.—*Cincinnati Medical News.*

Treatment of Paralysis Agitans.—The general tenor of experience in this and in kindred disorders is to the effect, (1) that the main indication is to nourish and support the failing power of the nervous centres affected; (2) that this is best accomplished by remedies drawn from the class of sedatives, or by the milder tonics. Henbane, conium, chloral, subcutaneous opiates, bromide of potassium, belladonna, hypophosphites or phosphorus, cod-liver oil, carbonate of iron, and sulphuret of potassium baths, with electricity in one or other of its three forms, appear to me the most hopeful remedies. But steady persistence in appropriate treatment is doubtless essential, and the want of this may account for many failures. Trousseau's adage should be borne in mind, "A longue maladie, longue traitement."—*Handfield Jones, British Medical Journal.*

Oxide of Zinc Ointment.—J. Kalish, in the *American Journal of Pharmacy*, suggests the following method of manipulation for preparing a smooth ointment of oxide of zinc: Rub the oxide in a wedgewood or unglazed porcelain mortar with considerable pressure, until as finely divided as possible, now add, gradually, with constant trituration and pressure, sufficient sweet oil of almonds to form a smooth paste; then add a little lard, mix thoroughly, and finally add the remainder of the lard. The same process will answer for all ointments containing insoluble substances.

Vehicle for Chloral Hydrate.—J. G. Plumer, in *London Pharm. Journ. and Trans.*, recommends, as the best vehicle for administering chloral hydrate, the sirupus flor. aurantii of the B. P. We have ourselves found nothing to answer better than essence of peppermint for disguising the peculiarly acrid and nauseous taste of the drug.

Acorn Coffee in Diarrhœa.—A German physician, Dr. Turk, says that after considerable experience he and many of his colleagues have arrived at the conclusion that, in cases of chronic catarrh of the bowels, drugs seldom produce the desired effect. A series of careful observations, however, have shown him that roasted acorns, prepared as coffee, with a few beans of the real article, form the best dietic, and at the same time medicinal remedy. Often when nitrate of silver, tannin, Dover's powder, etc., have proved useless, the simple acorn coffee (boiled, in cases of specially profuse diarrhœa, with from one to three grains of tannin, and in meteorism or sickness with the addition of a piece of orange peel to the decoction) has, from the first, lessened the stools and improved their quality, and very shortly restored appetite and nutrition. At the same time the children become not fat but healthy. The acorn coffee is more efficacious than alkalies, preparations of lime, tonics (Peruvian bark and extract) and carminatives. Moreover, the children drink it readily, without becoming tired, and the painful, formal and frequent administration of medicine is avoided.

Therapeutical Additions.—*L'Union Medicale* favorably reviews the addition to medical science during the past year, and cites in illustration of some of the most important: The action of belladonna against sweatings; the use of acetate of lead in pneumonia; electricity in the cure of Addison's disease; sulphate of copper in icthyosis. These remedies have not as yet been so largely tested in this country, and are worth consideration.—*Medical and Surgical Reporter.*

Ergot in Phthisical Hemoptysis.—Dr. Anstie, in the *Practitioner* for February, 1873, details some cases in which the fluid extract of ergot, in forty-minim doses, produced a marked effect in diminishing the amount of blood brought up by patients laboring under phthisis, in various stages. In his paper, which is to be continued in another number, he proposes to compare the benefits obtained from ergot with those from gallic acid, acetate of lead, digitalis, turpentine and alum.

Colorless Tincture of Iodine.—℞. Tincture of iodine, pure glycerin, aa. ℥j.; sulphite of soda, ʒj. Rub the salt to a powder in a small mortar, and add the glycerin gradually; then pour in the tincture of iodine, and triturate gently until a solution is effected, and the mixture assumes an amber color. It is asserted that the properties of iodine are increased by the addition of the sulphite of soda, and that the glycerin enhances the value and convenience of the preparation for local application.

TREATMENT OF GOITRE.—Professor Gross, in a clinical lecture, (*Philadelphia Medical Times*), says: When there is any heat in the part, my usual course is to apply leeches, and afterwards to use an ointment of the biniodide of mercury, which has been found a powerful sorbefacient; this, however, should not be applied just as it is dispensed in the shops, as it is much too strong. We will take one part of the ointment to eight of simple cerate, and of this will apply a small portion to the surface of the tumor daily, it being carefully washed previously.

Internally, we should expect the best results from some preparation of iodine; therefore, we shall give this patient liq. iodin. comp., gtt. x., three times daily, and at the same time admonish her to abstain from meats and live upon plain, mild diet.

In the cystic variety, the resolvent treatment is useless; here our only choice is either the trocar and the subsequent injection of iodine, or, with the knife and director, carefully to cut down, layer after layer, and enucleate the cyst. Extirpation of the gland has been practiced, but with rather unfavorable results. Dr. Maury, of this city, has twice performed the operation, once with entire success. When the gland is much enlarged, it must be a bold and fearless surgeon that would undertake such as operation, inasmuch as every step is accompanied with difficulties.

DR. BLEYNIE, *Revue Medicale de Toulouse*, recommends the use of phenic acid in puerperal fever epidemics, by having the lying-in chamber saturated with the odor and vapor, by means of cloths saturated with the solution and of open vessels, which would constantly diffuse it through the air. Doctors and midwives should also perfume themselves with it. Much better (says the Doctor) carry a disagreeable odor than the germs of a fatal malady.—*Medical and Surgical Reporter*.

TRIPLEX PILL OF DR. JOHN W. FRANCIS.—Take of powdered Socotrine aloes, powdered scammony, mercurial pill mass, aa. ʒj.; croton oil, gtt. xx.; oil of caraway, fl. ʒjss.; tr. aloes and myrrh, fl. ʒij. Mix the ingredients together by very thorough trituration, make a pill mass, and divide into four hundred pills. The usual aperient dose is one pill at bed-time till the natural condition is restored.—*Proc. Am. Pharm. Asso.*

ELONGATION OF THE UVULA.—Dr. F. B. A. Lewis, in the *Boston Medical and Surgical Journal* for April 13, records the case of a farmer, aged forty, in whom the snipping off of one-fourth of an inch of the elongated uvula gave immediate relief to a distressing irritative cough, which had interfered with his sleep and greatly impaired his general condition.

Treatment of Typhoid Fever.—The *Practitioner* for February quotes from the *Journal de Medicine* the following as the treatment of typhoid fever adopted by M. Pèter at La Charitè; Every other day he gives a glass of Seidlitz water, and morning and evening an emollient injection. The injection he considers to be useful in removing putrid matters. The glass of aperient fluid, without exhausting the patient like a daily purge, also keeps the intestinal canal clear from disintegrated matter. He uses the sulphate of quinine in quantities amounting to from seven to fifteen grains *per diem* as a febrifuge, increasing the dose in proportion to the intensity of the attack. He places great reliance on alcoholic stimulants, prescribing every day four or five ounces of quinine wine, which is made into a lemonade, so as to make from two to three pints of fluid, by which means the vegetable acids are freely and easily administered. These, he thinks, remove the crusts of the tongue, and the symptoms produced by putridity and relieved. He uses sponging with vinegar instead of baths or cold affusion, once daily.

Herpes Preputialis.—In one case of that character, with long prepuce, I endeavored to persuade a submittal to circumcision. He being unwilling, and believing it to be caused by moisture and acrid secretions from the mucous membrane, I caused him to roll the foreskin back, keeping dry by lint, and a double-tailed bandage, with result of complete healing and drying up of discharge. This is only one case, to be sure, but it had lasted upwards of two months.—*Dr. A. J. J., Medical and Surgical Journal.*

Sick Headache.—Dr. R. H. McKay, of Tennessee, writes us: For the benefit of Drs. Anstie, Wilks and others, who suffer with headache, I ask you to publish the following formula, the efficacy of which I have tested time and again: ℞. Granulated muriate of ammonia, one teaspoonful; morphiæ acet., gr. j.; water, ℔. ss. Sig. Dose for an adult, two teaspoonsful every ten minutes (precisely) till relief is obtained.—*Medical and Surgical Reporter.*

Cold Baths in Rheumatic Fever.—Dr. Sydney Ringer, in the *Practitioner*, reports the case of a girl, aged twenty-two, in whom rheumatic fever was treated by means of cold baths, and by the application of large-sized ice-bags, with evident benefit and an entirely successful result.

A Local Anæsthetic.—Albumen of egg, ℥j.; righolein, ʒiv.; oil of peppermint, ʒij.; collodion, chloroform, aa. ʒj. Mix. Agitate occasionally. Apply, by smart friction, with the hand, or apply with a camel's hair brush on the painful part.—*Medical Record.*

POST-PARTEM HEMORRHAGE.—Dr. Doyle related a case of post-partem hemorrhage, which he attended while a student. Pains were very slow, and scarcely perceptible at first. Upon examination he found the os dilated about one-half, and felt what he thought to be the head of the child, but afterward found it to be the bag of waters, with very firm membranes. He watched the case for three days, then attempted to rupture the membranes with the finger, but ineffectually, on account of their firmness; afterward succeeded with the scissors. The child was then expelled with a single pain. Immediate and copious hemorrhage followed. He applied ice-water over the abdomen, poured freely from a pitcher, and introduced a piece of the ice within the uterine cavity; the bleeding was arrested at once, the uterus contracting promptly and firmly.—*Lancet and Observer.*

DETERMINATION OF THE LIFE OR DEATH OF THE FŒTUS.—Dr. Cohnstein, in *Arch. fur Gynak.*, vol. iv., 3d part, 1872, (*Medical Record*, London), states that the information whether the fœtus is living or dead during pregnancy, but especially during parturition, is often of the greatest importance; and where hearing the fœtal heart and feeling the fœtal movements fail, or are uncertain, ascertaining the temperature *in utero* will often very materially assist if not decide us in determining the question. It is a fact that the temperature of the fœtus *in utero* is higher than the maternal temperature; and experience that the careful introduction of the thermometer into the uterine cavity, between the membranes and the wall of the uterus, is unattended by harm. We have thus a ready mode of settling the question when it is otherwise doubtful.

OPIUM ANTIDOTES.—Dr. H. G. Wallace (*Medical and Surgical Reporter*) says: "I see in the March 15th number of the *Reporter* an inquiry about antidotes. I agree that they are humbugs, but I will give my treatment in one case thought to be hopeless, in which I effected a complete cure. ℞. Potassium bromide, grs. viij.; Fougera's cod-liver oil, ℥ ss. S. Take three times a day. This effected a complete cure after taking about twenty bottles of the oil. He still continues the oil without the potash."

CHLORAL AFTER CHLOROFORM.—The editor of the *Canada Lancet* says that it is exceedingly unwise to administer chloral hydrate after an operation in which chloroform or ether has been used under any circumstances.

LOCAL STYPTIC.—I have been very successful with the use of permanganate of potassa as a local styptic. I use forty grains to the ounce of water.—*R. P. B.*, *Dental Cosmos.*

TOOTHACHE MIXTURE.—M. Trujillo, D.D.S., (*Dental Cosmos*), says: "The following mixture I have used for some time with the best results in toothache of children and adults. The mixture so combined is active, yet of an agreeable taste. ℞. Camphor, creosote, carbolic acid, aa. ℥ i.; sulph. ether, ℥ iss.; chloroform, ℥ ij.; sandarac gum, ℥ i.; Sydenham's laudanum, ʒ ij.; oil of cloves, ʒj. Mix first the camphor, carbolic acid and creosote till all the camphor is dissolved, and set aside. Next, mix separately sulph. ether and sandarac gum, forming a thick varnish when all the sandarac is dissolved; strain quickly through fine linen, avoiding all possible evaporation; mix with this varnish the laudanum, chloroform, the first mixture (camphor, carbolic acid and creosote), and add lastly the oil of cloves. Keep well corked for use. Clean and dry perfectly the cavity, and drop or two drops of the mixture into the cavity of the tooth, covering with a pledget of cotton. If relief is not obtained in a few minutes, repeat as before."—*Dental Cosmos.*

HYDRATE OF CHLORAL IN LABOR.—M. Bourdon, of Charité, has given chloral in ten cases in which the patients' strength was worn out before the last stage of labor, and when the pains had been severe for hours, the patients being irritated, etc., with the following results: The pains were diminished in intensity and frequency; in the intervals, the women were calm, even slept, and could thus repair, or at least direct their forces, while the labor became regular and of normal course. A remarkable fact to M. Bourdon was, that the uterine contractions, instead of losing their force, increased its intensity, so that the length of the labor was diminished, even in primaparas. Mr. Lambert, of Edinburg, calls attention to the same fact. The latter's conclusions are given, corresponding with the above.

Attention is called to the fact that deaths have been caused by chloral, so we should be on our guard in its administration.—*Clinic.*

GLEET AND CHRONIC IRRITATION OF THE URETHRA.—℞. Balsam copaiba, ℥j.; pulv. cubebs, ℥ss.; pulv. gum arabic, ℥j.; spts. nitre, ℥j.; sirup buchu, ℥iv.; cinnamon water to fill black bottle. Dose, two tablespoonsful twice a day.—*Chicago Medical Journal.*

FISTULA IN ANO.—Dr. Hute employs an ethereal solution of iodine in fistula in ano, which is more exciting than the tincture. Patients are not obliged to keep their beds. He has had several curses after one injection.—*Medical and Surgical Reporter.*

CUNDURANGO acts favorably in soft chancre (after cautery), and on the ulcers and eruptions of tertiary syphilis.—*Prof. Andrews.*

